# Domain-general demands that deactivate multiple-demand regions

**DOI:** 10.1093/cercor/bhag046

**Published:** 2026-04-22

**Authors:** Tamer Gezici, Elif Oymagil, Adem Yazici, Berhan F Akgur, Ipek Çiftçi, Ausaf A Farooqui

**Affiliations:** Department of Neuroscience, Bilkent University, Universiteler Mahallesi, 1606. Cadde, Çankaya, Ankara 06800, Turkiye; Interdepartmental Neuroscience Program, Bilkent University, Universiteler Mahallesi, 1606. Cadde, Çankaya, Ankara 06800, Turkiye; Aysel Sabuncu Brain Research Center, Bilkent University, Universiteler Mahallesi, 1606. Cadde, Çankaya, Ankara 06800, Turkiye; Department of Neuroscience, Bilkent University, Universiteler Mahallesi, 1606. Cadde, Çankaya, Ankara 06800, Turkiye; Interdepartmental Neuroscience Program, Bilkent University, Universiteler Mahallesi, 1606. Cadde, Çankaya, Ankara 06800, Turkiye; Aysel Sabuncu Brain Research Center, Bilkent University, Universiteler Mahallesi, 1606. Cadde, Çankaya, Ankara 06800, Turkiye; Aysel Sabuncu Brain Research Center, Bilkent University, Universiteler Mahallesi, 1606. Cadde, Çankaya, Ankara 06800, Turkiye; Department of Psychology, Bilkent University, Universiteler Mahallesi, 1598. Cadde, Çankaya, Ankara 06800, Turkiye; Department of Neuroscience, Bilkent University, Universiteler Mahallesi, 1606. Cadde, Çankaya, Ankara 06800, Turkiye; Interdepartmental Neuroscience Program, Bilkent University, Universiteler Mahallesi, 1606. Cadde, Çankaya, Ankara 06800, Turkiye; Aysel Sabuncu Brain Research Center, Bilkent University, Universiteler Mahallesi, 1606. Cadde, Çankaya, Ankara 06800, Turkiye; Department of Neuroscience, Bilkent University, Universiteler Mahallesi, 1606. Cadde, Çankaya, Ankara 06800, Turkiye; Interdepartmental Neuroscience Program, Bilkent University, Universiteler Mahallesi, 1606. Cadde, Çankaya, Ankara 06800, Turkiye; Aysel Sabuncu Brain Research Center, Bilkent University, Universiteler Mahallesi, 1606. Cadde, Çankaya, Ankara 06800, Turkiye; Department of Neuroscience, Bilkent University, Universiteler Mahallesi, 1606. Cadde, Çankaya, Ankara 06800, Turkiye; Aysel Sabuncu Brain Research Center, Bilkent University, Universiteler Mahallesi, 1606. Cadde, Çankaya, Ankara 06800, Turkiye; Department of Psychology, Bilkent University, Universiteler Mahallesi, 1598. Cadde, Çankaya, Ankara 06800, Turkiye; National Magnetic Resonance Research Center, Bilkent University, Universiteler Mahallesi, 1606. Cadde, Çankaya, Ankara 06800, Turkiye

**Keywords:** frontoparietal cortex, hierarchical control, metacontrol

## Abstract

Activation of multiple-demand regions during diverse difficult tasks has led to the recognition of domain-general demands that arise during any difficult task. Our tasks, however, are temporally structured episodes made of multiple components that are controlled as a single unit toward an overarching goal. Their numerous component control processes are organized through a goal-directed program instated at the episode’s onset. Difficult task episodes that require complex control processes also require a complex program to organize and coordinate them over time. Across four different fMRI experiments with different task designs and contents, we found that instituting these programs at the beginning of extended episodes involves a categorically different domain-general demand from that needed during their subsequent execution. This demand deactivated widespread regions, including the very same multiple-demand regions that activate during the execution of difficult tasks. The distinction between the demands related to program instatement versus those related to control during execution extended to psychophysiological signatures. In a fifth experiment, pupil diameter—typically increasing with control processes—decreased when more demanding programs were being instated. Instating overarching programs at the beginning of extended tasks thus constitutes a unique cognitive demand, distinct from the control processes engaged during task performance.

## Introduction

Neurocognitive accounts of goal-directed behavior have typically focused on control processes like attention, working memory (WM), response inhibition, configuring rule-related sets, etc. (Aron 2007; Botvinick et al. 2004; Corbetta and Shulman 2002; D’Esposito and Postle 2015; Duncan and Owen 2000; Fox et al. 2005b; Niendam et al. 2012; Posner and Rothbart 2007; Ptak 2012). These are processes that occur *during* task executions, and control ongoing processes in sensory and motor regions, and through these maintain the goal relevance of sensory and motor processing, filter irrelevant sensations, create correct stimulus–response configurations, maintain and update relevant representations in WM, select correct motor acts, etc. (Aron 2007; Desimone and Duncan 1995; D’Esposito and Postle 2015; Egner 2017; Gregoriou et al. 2009; Kastner and Ungerleider 2000; Miller and Cohen 2001). A foundational finding in this regard is that tasks that require more complex control processes show greater activation of a set of frontal and parietal regions, variously referred to as multiple demand (MD), attentional, or cognitive control networks, suggesting that any difficult task execution involves a common set of domain-general demands that are instantiated by the activation of this common set of brain regions (Assem et al. 2020; Duncan and Owen 2000; Fedorenko et al. 2013; Niendam et al. 2012b; Yeo et al. 2015). These MD activations are accompanied by the deactivation of a separate set of regions referred to as the default mode regions (DMN), which has been interpreted variously as the deactivation of regions involved in internal cognition, self-processing, or mind-wandering due to resources being directed to the current task execution (Raichle et al. 2001; Mason et al. 2007; Andrews-Hanna et al. 2014).

Tasks that require greater control demands are typically accompanied by the activation of modulatory cortical projections originating in brainstem regions, which, among other psychophysiological markers, correlate with increased pupil size (Kuipers et al. 2017; Makowski et al. 2020; Pourtois et al. 2020). Increased attention, WM load, rule-switching, response conflict, and perceptual complexity—all increase pupil size (Porter et al. 2007; Sara and Bouret 2012; Murphy et al. 2014; Joshi et al. 2016; Unsworth and Robison 2018; van der Wel and van Steenbergen 2018; Clewett et al. 2020). Projections from key MD regions—the frontal eye fields, the intraparietal sulcus, and the anterior cingulate—to the pretectal olivary nucleus, the superior colliculi, and the locus coeruleus have been hypothesized to cause this pupil dilation (Joshi et al. 2016; Strauch et al. 2022).

A feature of our cognition is that all our goals are achieved through extended, temporally structured task episodes that consist of numerous sequentially organized components, such as preparing breakfast, writing emails, and executing a block of trials. Executing extended tasks requires organizing and controlling the flow of cognition across the episode duration, ensuring that at every moment, it is proactively in the most optimal state for the demands expected at that moment. This requires both continuously bringing to the fore the relevant information, learnings, and procedures as well as continually making widespread attentional and set changes in the various neurocognitive domains. The various attentional, WM, and other control processes are not only to be made proactively at the moment when their demand is expected (Nobre and Stokes 2019; Nobre and van Ede 2023), but these different control interventions are made as part of a larger coordinated set of goal-directed changes being made across the episode duration. A 40-s-long task episode, for example, may require attention that is sustained for this duration; some junctures may require attending to parts of the environment, others may require preparing a motor act, and still others may require recalling an episodic memory, and so on. Furthermore, this attention being sustained may be in relation to something being maintained in WM and, at various junctures, may be guided through the coordinated recollection of numerous episodic memories and procedures (Logan 2002; Chun and Turk-Browne 2007; Taatgen 2013; Chen and Hutchinson 2019; Oberauer 2019). These numerous goal-directed changes made over the course of such episodes cannot be brought about by separate, independent cognitive acts every second or millisecond. They require a common program that subsumes task execution, creates and evolves the cognitive focus for relevant control operations, and organizes, instantiates, and maintains various control interventions.

Such programs would be the means through which intended goals organize and control the episode of cognition culminating in them and would be instated at the beginning of extended tasks (James 1890; Lewin 1926; Greenwald 1972; Jeannerod 1988; Gollwitzer and Sheeran 2006; Kruglanski and Kopetz 2009). Current accounts of task sets, focusing on single stimulus-to-response trials, recognize that task preparation requires recalling the relevant declarative representations and then transforming them into a procedural routine that involves a specific stimulus-to-response link that will be activated at the predicted instance (Logan and Gordon 2001; Brass et al. 2017; González-García et al. 2021). During extended tasks, a much more complex set of knowledge related to the episode will likewise be converted into a procedural routine. The target of this routine will not be limited to stimulus processing and motor responses but will include all relevant cognitive and control processes that need to be organized across the episode duration.

Beginning any extended task episode would therefore have the additional cognitive demand of instantiating these programs, with more difficult task episodes requiring more complex programs. Indeed, beginning any episode, regardless of its content, requires additional time, which correlates with the length and complexity of the ensuing episode (Cooper and Shallice 2000; Farooqui and Manly 2018b; Ruh et al. 2010; Schneider and Logan 2006). Thus, when motor sequences (eg finger taps, button presses for piano pieces, etc.) are executed, words/sentences are articulated, memorized lists are recalled, task lists are executed, even when episodes of activity corresponding to an uncertain number of unpredictable trials are executed—step 1 takes the longest to initiate, and this is longer for longer/complex episodes (Anderson and Matessa 1997; Farooqui and Manly 2018b; Henry and Rogers 1960; Kahana and Jacobs 2000; Klapp et al. 1973; Logan 2004; Poljac et al. 2009; Rosenbaum et al. 1983; Schneider and Logan 2006).

However, the cognitive demands of instantiating such programs at the beginning of extended tasks are likely to differ from those of control processes during their subsequent execution. Control processes are about selecting, maintaining, or updating the correct sensory or motor representations/processes or those linking the two. In contrast, instituting these programs requires assembling the set of commands that will go on to organize cognition and instantiate relevant control interventions *later* in the ensuing episode. While neural and psychophysiological accompaniments of control processes are well known, those of instantiating programs at the beginning of extended tasks are not. As we describe below, beginning extended tasks (and hence instantiating these programs) may entail a unique set of domain-general demands distinct from the well-characterized control processes.

### Beginning of task episodes involve unique, episode-related demands

Control demands (eg rule-switches, Stroop incongruence) elongate reaction times (RTs) and increase error rates. In contrast, step 1 of extended tasks, though showing longer RTs than subsequent steps, does not become more error-prone when there is no time constraint on responses. When participants were made to construe a flat run of trials as made of ~4-trial-long episodes, trial 1 had the longest RT. Crucially, while its RT was ~150 ms longer than subsequent trials, its error rates were not higher (Farooqui and Manly 2018b). In contrast, trials in the same study that involved rule-switching or Stroop incongruence were slower by only 50 to 100 ms but had significantly higher error rates, suggesting that the issue behind the elongated RT at trial 1 was distinct from those that elongated RT on rule-switching or Stroop trials.

Since these programs are related to the overarching episode, instating them at the beginning of task episodes involves creating a hierarchically organized cognition (Farooqui and Manly 2018b; Schneider and Logan 2006). An effect of this is that instating them at the beginning removes the rule-related switch cost at step 1. It is well known that changing rule-related configurations in response to a change in the relevant rule incurs a *cost*, slowing and making execution error-prone (Rogers and Monsell 1995). However, when rules change across consecutive steps crossing the boundaries of the overarching task episode, this switch cost is absent (Farooqui and Manly 2018b; Lien and Ruthruff 2004; Schneider and Logan 2015). This is because a change in the higher-level program at episode boundaries refreshes the lower-level, step-related rule configurations.

Lastly, and most relevant to the current study, while control processes have been linked to MD activations, the initiation of extended episodes (and hence the instating of programs) elicits deactivation across widespread regions, including MD regions (Farooqui and Manly 2018a). This deactivation is more intense at the beginning of longer episodes, paralleling the idea that longer episodes require a larger program. Likewise, cues that signal more demanding WM episodes deactivate MD regions compared to those that signal less demanding episodes (Manelis and Reder 2013).

### Will beginning difficult tasks deactivate MD regions?

The above raises a counterintuitive empirical possibility. If these programs, instated at the beginning, are the means of bringing about the various goal-directed control interventions across extended episodes, then difficult episodes that require a more complex set of control interventions will also require a more complex program. A task episode that (eg) requires a higher WM load and more complex updating will also require a more complex program to organize these WM interventions. If instating complex programs involves a distinct cognitive demand that deactivates MD regions, then MD regions would deactivate at the beginning of those same task episodes during whose subsequent execution they are well known to activate. The current study investigated this issue across four fMRI experiments.

The most reliable MD activations are observed in block designs with alternating episodes of hard and easy trials, irrespective of task content or modality (Fedorenko et al. 2013; Assem et al. 2020). The above predicts that the onset of difficult episodes would elicit a relative deactivation compared to the onset of easy episodes. Experiments 1 and 2 investigated whether such was the case. Experiment 3 compared the activity elicited at the beginning of difficult trial sequences that could only be executed as parts of an overarching episode against relatively easy trial sequences that could be executed as relatively independent units. If installing episode-related programs deactivates MD regions, then the former, requiring more complex programs, should deactivate them as well. The second part of this experiment investigated whether the beginnings of episodes that would subsequently involve greater working memory demands were associated with greater MD deactivation.

Experiment 4 uses a design in which the first step of demanding episodes also involves a greater working memory load. Since the latter is a well-known MD activator, this design pits the two tendencies—activation due to WM demands and deactivation due to programs instated at the beginning—against each other. If the latter were a stronger effect, MD regions would still deactivate at step 1 of demanding episodes even though participants were encoding a greater WM load at that time. Lastly, across these fMRI experiments, we investigated the behavior of DMN regions. Deactivation during difficult tasks is a well-known feature of the DMN. Would DMN deactivation be different at the beginning from that during the subsequent execution of the difficult task episodes? We had no hypothesis about this issue, but we nonetheless explored it across these experiments.

Finally, experiment 5 investigated the issue using pupil size. Any large-scale change in cortical activity is accompanied by changes in the modulatory brainstem systems that project diffusely to various cortical regions (Reimer et al. 2014). The activity of many of these systems (popularly adrenergic but also cholinergic, serotonergic, and even hypocretinergic) correlates with pupil size (Joshi et al. 2016). Control interventions and MD activations are typically accompanied by an increased pupil size (Mathôt 2018; van der Wel and van Steenbergen 2018; Strauch et al. 2022). If complex program instantiations at the beginning of difficult episodes deactivate MD regions, they may also cause a decrease in pupil size unlike the case with control processes well known to increase them.

## Materials and methods

### Experiments 1 and 2

**Figure 1:**
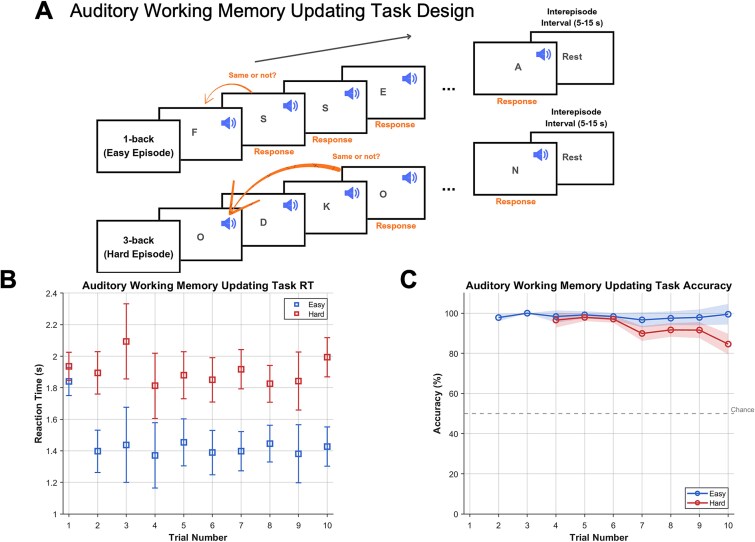
Experiment 1 (auditory working memory updating task): design and performance. (A) Participants performed an auditory working memory task alternating easy (1-back) and hard (3-back) blocks. On each trial, they heard a letter and indicated whether it matched the letter presented *n* trials earlier (1 trial for 1-back, 3 trials for 3-back). Each block consisted of 10 trials (only 5 are illustrated in the figure). Trials were self-paced; the next stimulus was presented 0.5 s after a button press response was made on the previous trial. The first 1-back trial and the first three 3-back trials did not involve a decision; on these trials, participants simply pressed any button to proceed to the next trial. Each block was followed by a 5- to 15-s rest period (inter-episode interval). (B) Mean reaction times (RTs) across trials for easy and hard blocks. Error bars represent 95% confidence intervals (corrected for within-subject comparison using the Cousineau–Morey method). (C) Mean accuracy across trials.

Twenty-four participants (13 females, mean age = 25.75 ± 5.8 yr) did an auditory working memory updating task, and 30 participants did a tactile decision-making task (19 females, mean age = 25.07 ± 5.4 yr). The auditory working memory updating task (experiment 1) was a modified auditory *n*-back task (Fig. 1). Easy and hard episodes involved 1- and 3-back tasks, respectively. Each episode had 10 trials. On each trial, participants heard a Turkish letter and responded whether the presented letter was the same or different from that presented *n* trials earlier (index finger: same; middle finger: different). The next trial started after a response was made, and the next trial stimulus followed the response to the previous one after 0.5 s. For the first 1-back trial and the first three 3-back trials, participants simply pressed a button to move on to the next trial since they did not have to make any comparisons on these trials and were only to keep the presented letters in their working memory.

Ten easy and 10 hard episodes alternated with each other with an inter-episode interval of 5 to 15 s. At the end of this interval, participants heard the identity of the next episode, along with instructions to either continue resting or press a button to start the next episode whenever they were ready. Trial 1 stimuli came up immediately after this button press. Participants completed a 10-min pre-scan training before the fMRI session. For 3 participants whose responses remained erroneous at the end of this 10-min session, the hard episodes were changed to 2-back. The experiment was delivered via Psychtoolbox in MATLAB. Auditory stimuli and prerecorded instructions were delivered through MR-compatible pneumatic headphones manufactured by Troyka Med (Ankara, Turkey). The system involved a mono loudspeaker positioned inside the scanner room within an radio frequency-shielded enclosure; sound from this loudspeaker was transmitted to the participant’s ears via pneumatic air-tubing connected to the headphones. The headphones included foam padding that fit snugly around the ears and provided substantial passive attenuation of scanner noise. For each participant, stimulus intensity was set to a comfortable volume prior to scanning, ensuring clear audibility throughout the tasks.

In the tactile decision-making task (experiment 2), participants made judgments about the size of geometric shapes (eg circles, squares, and rectangles) embossed on a plexiglass tablet placed on the participant’s chest or abdomen. Each tablet was bisected horizontally into an easy section and a hard section through three raised lines running through the middle. This helped participants discern the boundary between the two sections through touch. Each section was further divided into five cells by four vertical lines spaced 3 cm apart. These cells represented individual trials and contained a pair of vertically aligned shapes drawn by raised lines. A tablet thus consisted of an easy episode and a hard episode, each comprising five trials. Participants executed 10 easy and 10 hard episodes on 10 tablets.

The pair of shapes presented in a single trial was drawn 2 cm apart; they were always identical but differed in size. Shapes varied across different trials. On each trial, participants had to decide which of the two was larger and convey their answer by pressing a button (index finger for the top shape; middle finger for the bottom shape). They were then cued auditorily to move on to the next trial. This cue followed the response to the previous trial by 0.5 s. After a block of five trials, a jittered rest period of 5 to 15 s followed. The decision difficulty was manipulated by differences in the sizes of shapes in a pair and by the extent to which their margins were raised. For easy trials, the difference in size between the shapes of a pair was between 0.8 and 1 cm^2^. For hard trials, this difference ranged from 0.3 to 0.5 cm^2^. The extent to which the margins of the shapes were raised was lower in hard blocks (0.5 mm vs. 1 mm), making tactile judgments more demanding.

Numerical indicators for easy (1) and hard (2) were embossed on the upper left edge of their respective parts. These not only informed participants about the nature of the ensuing episode but also served as a tactile reference point, orienting them to where to start their block of trials. After every inter-episode interval, participants were asked to locate the tactile reference point of the next episode and press a button, eg “please spot the beginning of the hard block by placing your finger on the corresponding marker, then press a button when you are ready to proceed with trial 1.” Immediately after response to a trial, they heard an instruction asking them to locate the trial 2 stimulus and press a button. After this button press, participants proceeded to make their decision about trial 2 and respond. They likewise proceeded with trials 3, 4, and 5. After the response to the fifth trial, they were told to rest until they heard instructions about the next episode. After the rest, they would then hear: “Please spot the beginning of the easy block by placing your finger on the corresponding marker, then press a button when you are ready to proceed with trial 1.”

Since a single tablet lasted only two episodes, it had to be changed after each pair. This was done after the jittered rest period at the end of every second episode. Participants would pick a new tablet from the stack of unused tablets on one side, place it on their chest/abdomen, and return it to the other side after completing it. This too was coordinated via auditory instructions, eg “please remove the current tablet and pick the next one, and locate the beginning of the hard block by placing your finger on the corresponding marker, then press a button when you are ready to proceed with trial 1.” The order of hard and easy episodes on a tablet was counter-balanced across participants. Prerecorded auditory instructions were delivered via Psychtoolbox in MATLAB, conveying instructions to participants.

#### Neuroimaging analysis

Each task episode was modeled using three regressors. The start of the episode, corresponding to the button press participants made to start the episode, was modeled as an event of no duration to capture the activity elicited by the beginning of the episode. The entire episode was separately modeled as an epoch of the same duration as the episode to capture the average activity elicited during the episode. The completion of the episode was modeled as an event of no duration to capture completion-related activity. The event regressor, modeling the beginning of the episode, and the epoch regressor, modeling its duration, though consecutive with each other, had low collinearity (experiment 1: 0.22 to 0.24; experiment 2: 0.13 to 0.15), allowing us to disentangle the phasic activity elicited at the beginning from the sustained activity elicited across the episode’s duration. The easy and hard episodes were separately modeled with a different set of regressors. Movement parameters were added to the GLM as covariates of no interest. Regressors and covariates were convolved with the standard hemodynamic response function and entered into the GLM. We did two t-contrasts. The first looked at hard > easy at the beginning of the episodes. The second looked at hard > easy during the main episode epoch. Contrast estimates from each participant were entered into a group-level analysis. We then did a whole-brain repeated-measures ANOVA to look for regions where the effect of these two contrasts differed. The resulting maps were rendered with MRIcroGL on the MNI152 template.

### Experiment 3

Three groups of participants were involved. Group 1 executed a loosely organized sequence of nine 3-back trials. Group 2 executed a well-defined task episode in which nine similar trials served as steps toward a defined goal—counting the total number of 3-back repeats. Group 3 performed a task identical to that of group 2 but less demanding. It involved counting the number of 2-back repeats across six trials.

#### Group 1

Participants (29 participants, 18 females, 11 males; age range 18 to 28 yr, mean age 21.4 ± 2.4 yr) saw a picture on every trial and had to respond if the current picture was the same as three trials earlier (index-finger button: same; middle-finger: different). The picture remained on until a response was made, and the next picture appeared after a jittered interval of 1 to 8 s (average 3.8 s). Nine or 6 trials made a sequence. Trial sequences began with a start-screen that remained on until a button was pressed and informed whether the ensuing sequence would be 9 or 6 trials long; trial 1 of the ensuing sequence started 1 to 8 s after this button press. At the end of each sequence, participants were presented with feedback (displayed for 1 s) on their performance. If they had no errors, their score increased by 1; if they had made only one error, their score remained unchanged; two or more errors decreased their score by 1. The feedback screen displayed the change in score and the current score. The next start-screen came 2 to 10 s after the feedback on the previous sequence. A fixation cross was presented at the center of the screen during inter-trial and inter-sequence intervals. Pictures were taken from a pool of 89 colorful pictures.

Before the actual experiment session, participants practiced the task until they had fully grasped the rules and could execute at least two consecutive sequences without errors. Seventeen participants executed 2 runs, 11 executed 3, and 2 executed 4. Each run consisted of 16 sequences, equally divided into short (ie six trials) and long (ie nine trials) types. Note that in this article, we report only the results of nine trial sequences This experiment was originally designed for a different study (Giray et al. 2026).

#### Groups 2 and 3

Participants performed a modified visual *n*-back task. Fourteen participants completed the 3-back task (group 2; 9 females, 5 males; mean age 22.6 ± 3.41 yr), and 17 participants (group 3; 9 females, 8 males; mean age 22.9 ± 3.46 yr) completed the 2-back task. The task was organized into episodes consisting of 6 trials for the 2-back and 9 for the 3-back versions. Each episode began with a start-screen that remained on until a button was pressed. Trial 1 started after 1 to 8 s. Each trial involved a picture presentation. Unlike the usual *n*-back tasks, participants covertly compared the current picture to the one presented two trials earlier in the 2-back task (and three trials earlier in the 3-back task), and counted repeats across the episode, reporting the total at the end. They could see up to 3 repeats in a sequence. After participants reported their count, feedback was provided, including the score for the current episode (+1 for a correct response, −1 for an incorrect response) and their total score (eg total score = 5; last change = −1). The inter-episode interval was jittered between 2 and 15 s. Pictures were presented for 1 and 1.5 s for 2-back and 3-back, respectively, with an inter-trial interval jittered between 1 and 8 s (average 3.8 s). There was no interval after the final trial; participants were immediately presented with the probe screen. Stimuli were colorful pictures selected from sets of 16 for the 2-back (group 3) and 22 for the 3-back (group 2) episodes. Prior to scanning, participants completed a 10-min practice block outside the scanner. The main experiment comprised four functional runs, each containing 20 sequences for the 2-back task and 16 sequences for the 3-back task. For all groups, the experiment was delivered through Psychtoolbox on MATLAB.

#### Neuroimaging analyses

To estimate the time series of activity starting from the beginning of the trial sequences without assuming a canonical hemodynamic response function (HRF), we modeled the BOLD signal from the onset of the first trial using a set of 2-s-long finite-impulse regressors (FIRs) (Glover 1999). The duration modeled was 50 s in group 1 (25 FIR regressors), 36 s in group 2 (18 FIR regressors), and 26 s in group 3 (13 FIR regressors). Window length was set according to the mean duration of the sequence (from the first trial’s onset to the last trial’s offset). Individual trials (barring the first trial), probe, feedback, and the start-screen were separately modeled, and their regressors were convolved with the canonical HRF. Trials were modeled as epochs of duration equal to the time the stimulus remained on. This would be the duration of RT for group 1, 1.5 s for group 2, and 1 s for group 3. The first trial was omitted from HRF modeling because it was being modeled by FIR regressors. The start-screen was modeled as an epoch lasting for the participant’s response time. This allowed us to estimate the time series of activity starting at trial 1 and continuing across the episode after estimates of activity elicited by subsequent trials had been accounted for. Movement parameters were included in the model as covariates of no interest.

Beta estimates from the first 13 FIR time bins (covering the initial 26-s period after the first trial onset) were plotted as a line graph with a ribbon showing the 95% confidence interval that was adjusted for within-subject comparisons across time bins. As an aid to visual comparison, the first time bin’s beta estimate of each line plot was aligned to zero, and the subsequent time bins were adjusted accordingly. This adjustment was performed solely for plotting purposes; all statistical analyses were conducted on the original beta estimates.

We tested whether the time course of activity differed between (i) group 1 and group 2 and (ii) group 2 and group 3 using a Bayesian mixed-effects ANOVA implemented in R (BayesFactor package, function ‘anovaBF’). Fixed factors were group, time, and ROI, and subject was entered as a random factor. The model included all main effects and interactions. Default Jeffreys–Zellner–Siow priors (scale = 0.5) were used. After fitting the full model, we obtained inclusion Bayes factors (BF\_incl) for each effect with ‘bayesfactor_inclusion’ (‘match_models = TRUE’). We also conducted a whole-brain mixed ANOVA that looked at regions where the change in activity across the FIR bins was different across groups 1 and 2, and across groups 2 and 3.

### Experiment 4

Thirty participants (20 females, mean age = 21.5 ± 2.4 yr) executed task episodes requiring them to remember the orientations of two distinct sets of lines across two subtasks. These episodes could either be hard or easy. Episodes began with a start-screen that remained on until participants pressed a button, followed by step 1 (subtask B-line presentation), during which either a single line (easy episodes) or two lines (hard episodes) were presented. These lines were presented around the center at a visual angle of 2° from the center and were separated by at least 90°. The length of these lines was 1.5°, and the thickness was 0.1°. The lines could be of one of four colors (red, green, blue, or white). The two lines presented during hard episodes were never of the same color. Their orientations could vary from 0° to 179°. The screen background was gray (RGB = 0.5, 0.5, 0.5). These lines stayed on until participants pressed a button. Immediately at the offset of these lines, a mask screen was presented for 500 ms. The mask was composed of randomly oriented lines of the same color as the stimulus lines. Step 1 was followed by step 2 (subtask A-line presentation), whereby two lines of different colors were presented. These, too, remained until a button was pressed, and a mask screen followed their offset, staying on for 500 ms.

Participants were then probed to recall one of these A-line orientations (step 3). The probe for the line orientation to be recalled was presented at the same spot and in the same color as the original line. Participants changed the probe orientation by repeatedly pressing a button. Index- and middle-finger presses rotated the orientation clockwise and anticlockwise, respectively, each press changing it by 1°. After participants were satisfied with the probe orientation, they made a separate button press to confirm their answer. This removed the probe and displayed the feedback, which was the angular deviation between the actual orientation and their answer. Lastly (step 4), they recalled the orientation of one of the B lines presented in step 1. The probe again was of the same color and at the same spot as the line to be recalled. The probe manipulation was identical to that at step 3. This step, too, terminated with the confirmatory button press and was followed by feedback indicating the angular deviation between their answer and the original orientation. The start-screen for the next episode followed step 4. Each step was separated by a jittered gap of 0.5 to 6 s, calculated from the offset of the previous step to the onset of the next.

Participants completed 20 easy and 20 hard episodes, which alternated. The order reset after every 4 episodes, eg for the first four episodes, the order may be easy-hard-easy-hard. For the next four, the order may shift to hard-easy-hard-easy. Data were collected across two separate fMRI runs, each including 10 easy and 10 hard episodes and lasting 15 to 25 min.

#### Neuroimaging analyses

To capture the activity linked to each of our events of interest without making hemodynamic assumptions, we modeled 12 s of activity succeeding the beginning of each of the four steps using six 2-s-long finite-impulse regressors. Steps related to easy and hard episodes were separately modeled. Movement parameters were added to the GLM as covariates of no interest. The collinearity between the FIR regressors from steps 1 and 2 was very low. The highest collinearity was between the sixth regressor (modeling step 1) and the first regressor (modeling step 2), but even this was 0.1 or less. Collinearity among the other regressors ranged from 0.01 to 0.08. We conducted a repeated-measures ANOVA to identify regions where the average activity across the six FIR bins for hard and easy episodes differed between steps 1 and 2. Bayesian ROI analyses were done in the same way as experiment 3.

### Experiment 5

The design of the experiment was identical to experiment 1, except that a jittered gap was no longer used to separate the component steps; instead, they were separated by a constant 1-s gap. Thirty participants (20 females, mean age = 20.6 ± 1.8 yr) were tested. Stimuli were delivered in a dark room on a computer monitor (NEC MultiSync LCD 2190UXP, 21 in., resolution: 1600 × 1200, refresh rate: 60 Hz) placed 57 cm from participants, whose heads were fixed and chins resting. Pupil sizes were recorded using Eye Tracker 6 (Applied Science Laboratories, Bedford, MA, USA) at a sampling rate of 50 Hz. Participants responded via arrow keys on a QWERTY keyboard.

The width and color of lines (same as experiment 4) were chosen such that easy and hard screens were iso-luminant (https://www.w3.org/Graphics/Color/sRGB). In-house-developed Python scripts were used to analyze pupil-size data. NaNs replaced eye-blink values. Extreme pupil sizes were discarded using a cutoff value of 4 SD above and below the median pupil diameter for each subject. We did not apply any smoothing to the data. The timing of pupillometric and behavioral outputs was aligned at the beginning by a marker sent from the experiment script to the eye tracker via the XDAT port. Each of the four steps of an episode ended with a button press. For each step, we took the time series of pupil sizes over the preceding and succeeding 1 s of the button press. Experiments 4 and 5 were run using PsychoPy version 2021 February 3.

### MR acquisition and preprocessing

MRI scans were acquired on a 3-Tesla Siemens MAGNETOM Tim Trio scanner with 12-channel (in experiments 1 and 2) and 32-channel (in experiments 3 and 4) phased array head coils. Stimuli were displayed via an MR-compatible 32-in. LCD display (60 Hz refresh rate; 1280 × 768 pixels). Responses were collected via a fiber-optic button box (Current Designs, fORP 904 fMRI trigger box and response system). A sequential descending T2*-weighted gradient-echo planar imaging (EPI) acquisition sequence was used with the following parameters: acquisition time of 2000 ms, echo time of 30 ms, 32 oblique slices with a slice thickness of 3 mm and a 0.75-mm interslice gap, in-plane resolution of 3.0 × 3.0 mm^2^, matrix dimensions of 64 × 64, a field of view measuring 192 mm, and a flip angle of 78°. T1-weighted MPRAGE structural images were acquired for all subjects, characterized by a slice thickness of 1.0 mm, resolution of 1.0 × 1.0 × 1.0 mm^3^ isometric voxels, a field of view spanning 256 mm, and 176 slices. Experiments 3 and 4 used GRAPPA and 20% phase oversampling with an acceleration factor of 2; these methods were not used in experiments 1 and 2.

Preprocessing and subsequent GLMs were conducted using Statistical Parametric Mapping (SPM12) via the automatic analysis (aa) pipeline, version 5 (Cusack et al. 2015), in MATLAB. During preprocessing, functional images were slice-time corrected using the middle slice as a reference, co-registered with the structural T1-weighted images, and then realigned and normalized to the Montreal Neurological Institute (MNI) template. Normalization was performed using the DARTEL toolbox in SPM. EPI images were then resampled to a 2 × 2 × 2 mm^3^ resolution and subsequently smoothed using an 8-mm full-width at half-maximum Gaussian kernel. The time course of each voxel was high-pass filtered with a 128-s cutoff period, and movement parameters were included in the model as covariates of no interest.

### Regions of interest

All of our fMRI results are evident in whole-brain analyses. Our main reason for doing ROI analyses was to display the pattern of activities in key MD and DMN regions. We used mask MD ROIs taken from Fedorenko et al. (2013). These included anterior middle frontal gyrus (aMFG), middle middle frontal gyrus (mMFG), posterior middle frontal gyrus (pMFG), frontal eye fields (FEF), pre-supplementary motor area (preSMA), intraparietal sulcus (IPS), and anterior insula (AI). DMN ROIs—posterior cingulate cortex (PCC), anteromedial prefrontal cortex (aMPFC), and temporoparietal junctions (TPJ)—were taken from Andrews-Hanna et al. (2010). All ROIs were in MNI space. ROIs were made by using the MarsbaR toolbox for SPM. ROI analyses were also done using this toolbox.

### Participants

All participants were in the 18-to-43-yr age range. All gave their written consent prior to participation. The Research Ethics Committee of Bilkent University approved all experimental protocols and procedures (Approval number: 2020_01_27_04). All data collection occurred at the National Magnetic Resonance Research Center (UMRAM) in Ankara.

## Results

Contrasting hard versus easy episodes is well known to increase activation in MD regions. The most reliable source of this finding is block-design studies with alternating blocks of hard and easy conditions. In experiments 1 and 2, we therefore used the same design and investigated whether MD regions deactivated at the beginning of those very same hard episodes during whose subsequent execution they are well known to activate.

### Experiment 1

Participants performed hard and easy episodes (each consisting of 10 trials) of an auditory *n*-back task, which involved maintaining and updating either 3 items (hard episodes) or 1 item (easy episodes) (Fig. 1). They waited for roughly the same duration before pressing the start button and beginning easy or hard episodes (easy: 4.41 s; hard: 4.48 s, *t*_23_ = 0.05; *P* = 0.97). They were expectedly slower and more erroneous during 3-back compared to 1-back episodes (Fig. 1; *F*_1,20_ > 56.682, *P* < 0.001 for both RTs and accuracies).

**Figure 2:**
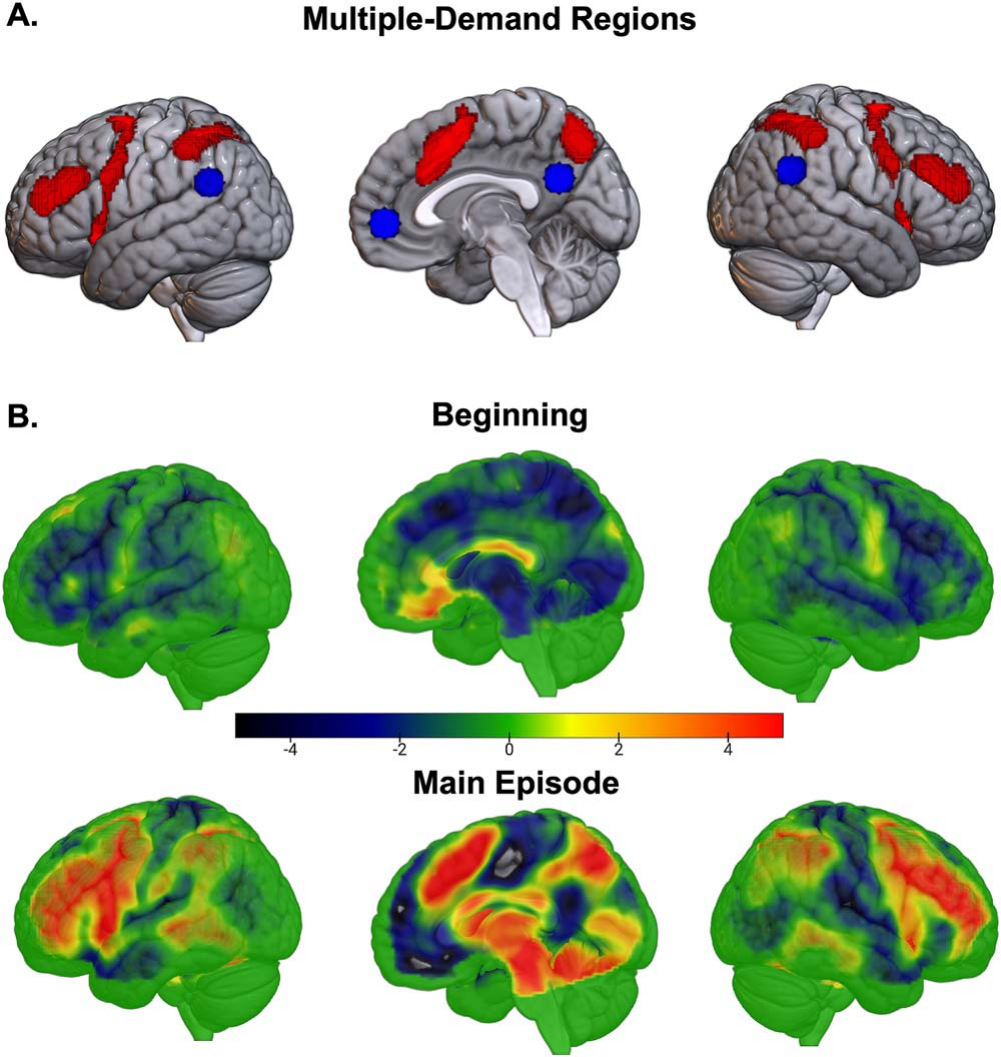
(A) Multiple-demand regions (red) include the middle frontal gyrus, frontal eye fields, anterior insula, pre-supplementary motor area, and intraparietal sulcus. We also analyzed key nodes of the default mode network (blue): anteromedial prefrontal cortex, posterior cingulate, and temporoparietal junctions. (B) (Experiment 1) T-maps of the contrast hard > easy at the beginning and during the main episode. MD regions activated more for hard episodes only during the main episode epoch. At the beginning of the episode, MD regions, along with other regions, decreased their activity for hard episodes. The t-threshold corresponding to an FDR correction at *P* <.05 was at 2.4 and 2.2 for the top and bottom images.

Our primary aim was to compare hard and easy episodes in terms of the activity elicited at their beginning versus that elicited during the execution of the main episode. We first show unthresholded t-maps so that regions that show trends but do not reach statistical significance also get displayed. Figure 2 shows regions where activity at the beginning and during the execution of hard episodes was higher (hot colors) and lower (cold colors) than the corresponding parts of easy episodes. As evident, the beginning of hard compared to easy episodes resulted in a deactivation in widespread brain regions, including MD regions. In contrast, the activity during the subsequent execution of the hard episode was higher in these same MD regions.

**Figure 3:**
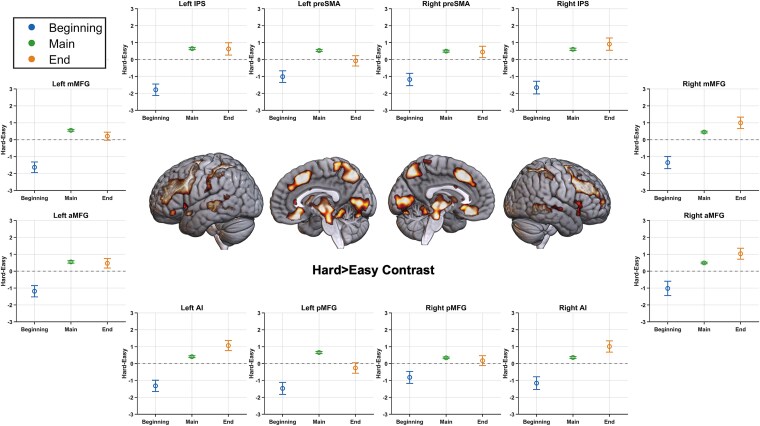
(Experiment 1) Brain render shows regions where the effect of difficulty was different at the beginning compared to during the main episode (FDR, *P* < .05). Plots show the difference between activities during hard and easy episodes at their beginning, during the main episode duration, and at their end in the different MD regions. In all of them, the beginning of hard episodes elicited lower activity than the beginning of easy episodes, creating a negative hard-easy difference; however, these same regions activated more during the subsequent execution as well as at completion of these hard episodes. Error bars represent 95% confidence intervals. ROIs include anterior middle frontal gyrus (aMFG), middle middle frontal gyrus (mMFG), posterior middle frontal gyrus (pMFG), pre-supplementary motor area (preSMA), intraparietal sulcus (IPS), and anterior insula (AI).

A whole-brain repeated-measures ANOVA (Fig. 3) looking for this differential response to difficulty at the beginning versus during the subsequent execution of the episode showed significance at all MD regions—bilateral middle frontal gyrus extending up to the anterior prefrontal cortex, anterior insula, intraparietal sulcus, and pre-supplementary motor area. As evident in the plots of these regions, across all regions, hard episodes showed stronger deactivation at the beginning but stronger activation during subsequent execution. Combined across all MD ROIs, a Bayesian repeated-measures ANOVA examining the difference between the two phases (beginning and main episode) in the effect of difficulty (hard–easy) was highly significant (BF_incl_ = 10^6^). Lastly, the completion of hard episodes elicited greater activation than that of easy episodes, perhaps reflecting the dismantling of more complex programs at the completion of hard episodes (combined across MD ROIs; BF_10_ = 10^10^).

### Experiment 2

**Figure 4:**
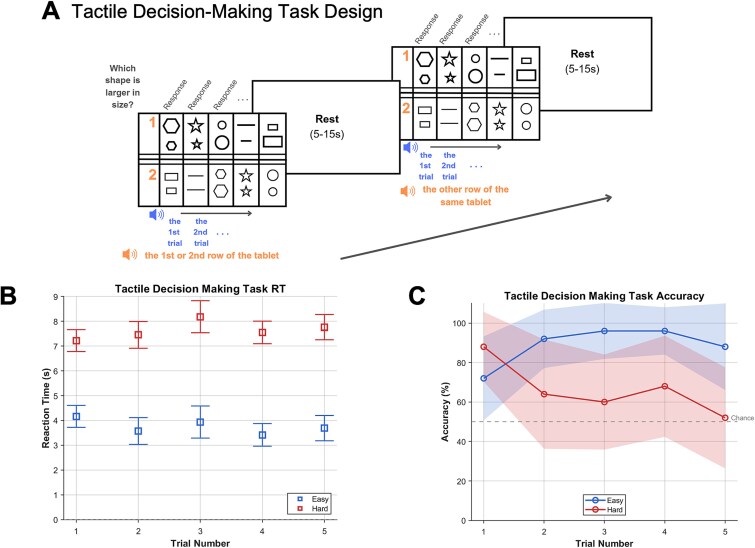
(Experiment 2) Tactile decision-making: participants decided which of the two shapes was larger in size. The task was done through a plexiglass tablet that had an easy and a hard part, with 5 trials each. Each trial involved a pair of shapes drawn with raised margins. Margins of shapes on easy trials were raised more above the surface, making them easier to perceive by touch. Furthermore, the size difference between the shapes of a pair was larger on easy trials. The completion of each episode was followed by a 5- to 15-s rest period (inter-episode interval). (B) Reaction times (RTs) and (C) accuracy for easy and hard blocks across trials. Error bars and shaded margins represent mean 95% confidence intervals.

Experiment 2, which had a very different task structure and content, showed the same findings, albeit with weaker effect sizes. Here, participants executed hard and easy episodes involving 5 tactile judgment trials (Fig. 4). Participants waited for roughly equal lengths of time before starting hard or easy episodes (7.7 vs. 7.2 s, *t*_29_ = 1.5, *P* = .13). Behaviorally, they were slower and more erroneous during hard episodes (*F*_1,20_ > 2603, *P* < .001, for both RT and accuracies; Fig. 4). We again compared the hard and easy episodes in terms of the phasic activities elicited at their beginnings (to capture the activity related to program instantiation) and sustained activity elicited during their executions (to capture activity related to the control processes made during their executions), as well as phasic activity elicited at their completions.

**Figure 5:**
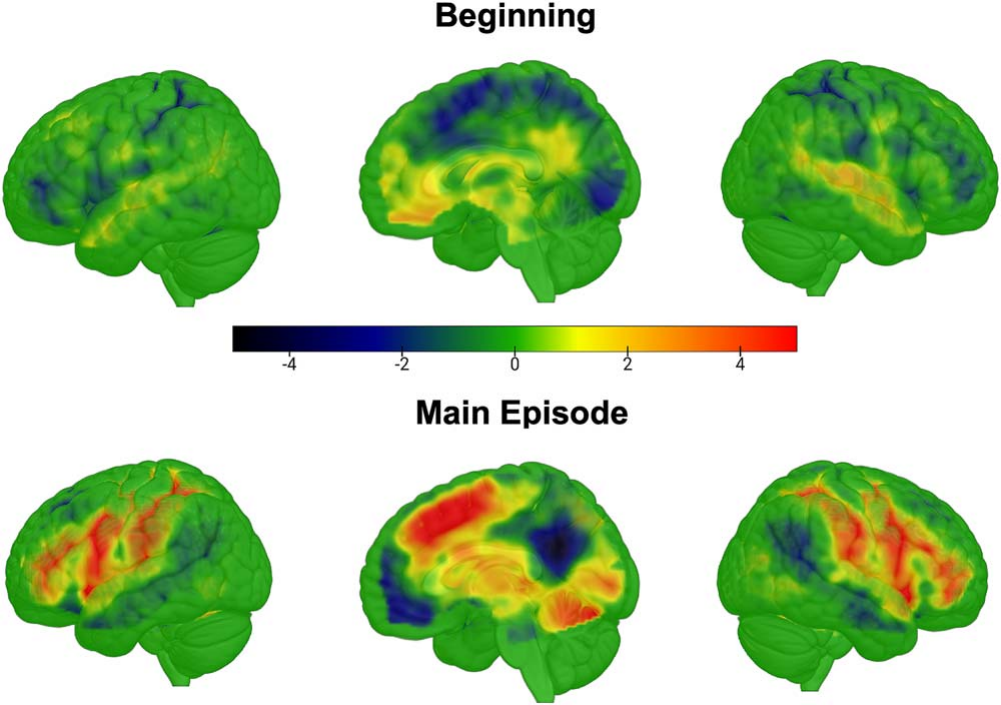
(Experiment 2) Unthresholded t-maps of the contrast hard > easy at the beginning and during the main episode. As before, MD regions showed greater activation for hard episodes only during the main epoch. At the beginning of the episodes, hard episodes produced a deactivation in certain MD regions, such as the preSMA and right IPS, compared with easy episodes; however, these effects did not reach significance after FDR correction at *P* <.05. Importantly, no MD region showed higher activation at the beginning of hard episodes. During the main episode period, by contrast, hard episodes elicited increased activation across all MD regions. The t-threshold corresponding to an FDR correction at *P* <.05 for the top (beginning) image was at infinity (due to very few clusters showing any significant effect) and 2.2 for the bottom image.

**Figure 6:**
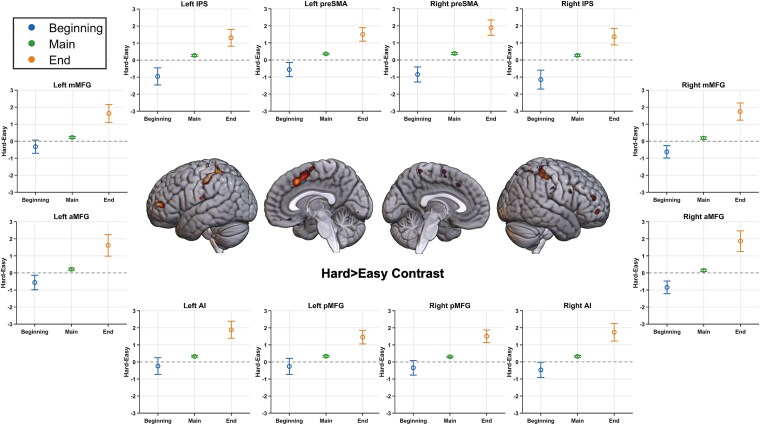
(Experiment 2) Brain render shows regions where the effect of difficulty was different at the beginning compared to during the main episode (uncorrected *P* < .001). Plots show the difference between activities during hard and easy episodes at the beginning, during the main episode duration, and at completion in the different MD regions. In all of them, the beginning of hard episodes elicited lower activity than the beginning of easy episodes, creating a negative hard–easy difference; however, these same regions activated more during the subsequent execution as well as at completion of these hard episodes. Error bars represent 95% confidence intervals. ROIs include anterior middle frontal gyrus (aMFG), middle middle frontal gyrus (mMFG), posterior middle frontal gyrus (pMFG), pre-supplementary motor area (preSMA), intraparietal sulcus (IPS), and anterior insula (AI).

Figure 5 shows regions where activity at the beginning and during the execution of hard episodes was higher (hot colors) and lower (cold colors) than the corresponding parts of easy episodes. Increased MD activation during hard episodes was again limited to the main episode and did not occur at the beginning. While the onset of hard episodes did not result in widespread deactivation (unlike experiment 1), key MD regions (preSMA, IPS) showed a trend toward deactivation. The differential effect of difficulty at the beginning versus during the execution of the episode failed to reach significance at the whole-brain level with repeated-measures ANOVA when corrected for multiple comparisons using the false discovery rate (Fig. 6). In the ROI analysis, a Bayesian repeated-measures ANOVA examining differences in the effects of difficulty across two phases (beginning vs. main episode) failed to reach significance when combined across all ROIs (BF_10_ = 1.5). A post hoc analysis, however, showed that the hard–easy difference was significantly negative at the beginning (BF_10_ = 75,282) and significantly positive during the main episode (BF_10_ = 10^20^). Lastly, as in experiment 1, completion of hard episodes elicited stronger activation than that of easy episodes (BF_10_ = 10^52^).

**Figure 7:**
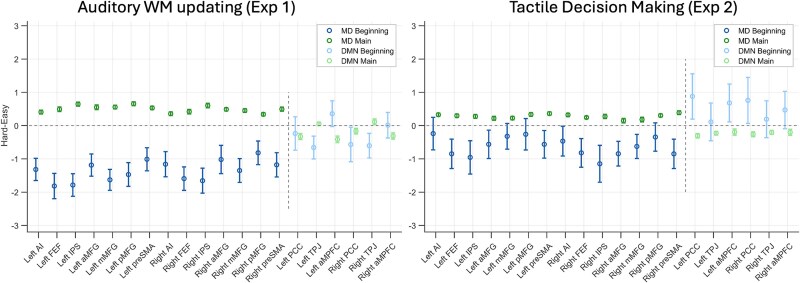
While MD regions deactivated to difficulty at the beginning of episodes and activated later during its main execution across both experiments 1 and 2, DMN regions’ response was inconsistent across these experiments. Error bars represent 95% confidence intervals. MD ROIs include anterior middle frontal gyrus (aMFG), middle middle frontal gyrus (mMFG), posterior middle frontal gyrus (pMFG), frontal eye fields (FEF), pre-supplementary motor area (preSMA), intraparietal sulcus (IPS), and anterior insula (AI). DMN ROIs include posterior cingulate cortex (PCC), anteromedial prefrontal cortex (aMPFC), and temporoparietal junctions (TPJ).

Across the two experiments above, MD regions deactivated at the beginning of hard episodes compared to easy ones but activated later during their subsequent executions. This effect was shown by all MD regions. We specifically looked for distinctions between the cingulo-opercular (anterior insula and pre-supplementary motor areas) and the frontoparietal subgroups (middle frontal gyrus and intraparietal sulcus), regions within the MD set that have been considered to be functionally distinct (Dosenbach et al. 2006). However, the behavior of these sets of regions did not differ (BF_incl_ = 1). We also examined the behavior of DMN regions, which are well known to deactivate during hard episodes. As shown in Fig. 7, their response to load at different phases of task episodes was very different from that of the MD regions. While they deactivated to difficulty during the main episode, their response to difficulty at the episode beginning was inconsistent. In experiment 1 (auditory WM updating task), they mostly deactivated at the beginning of hard episodes, but in experiment 2, they mostly activated.

Two potential issues can be raised about experiments 1 and 2. First, since they had alternating hard and easy episodes, perhaps activity elicited at the end of the previous (eg hard) episode got smeared into the activity at the beginning of the next (eg easy) episode. Second, since the episodes were modeled with three regressors—two event regressors of no duration, modeling the beginning and the end, and an epoch regressor modeling the episode duration—perhaps the sluggishness of the BOLD response and the collinearity among these regressors, in some way, affected the findings. Both objections are unlikely.

First, completions of episodes were modeled with separate regressors, making it unlikely that completion activity will get captured by the start regressor of the next episode, separated by a jittered interval of at least 5 to 15 s (since participants would frequently rest for a couple of seconds before starting the next episode, the actual intervals were larger). Explicitly modeling the intervening rest period (done via a separate GLM) did not change the results. Regarding the second issue, while the collinearity between the beginning and the main-task episode regressors was low in both experiments (~0.14 and ~0.22), to rule it out further, we conducted another analysis.

In a new analysis, we modeled only the beginning and the end of the episodes using two event regressors of no duration, and left the main episode duration unmodeled. If the relative deactivation at the beginning of hard episodes was an artifact due to the simultaneous modeling of the episode by an event regressor at the beginning and an epoch regressor for the duration, then it should be absent when there is no epoch regressor. In fact, if, as predicted by existing accounts, the beginning of hard episodes activated MD regions, then in this new analysis, the beginnings of hard episodes should have higher activation than easy episodes.

**Figure 8:**
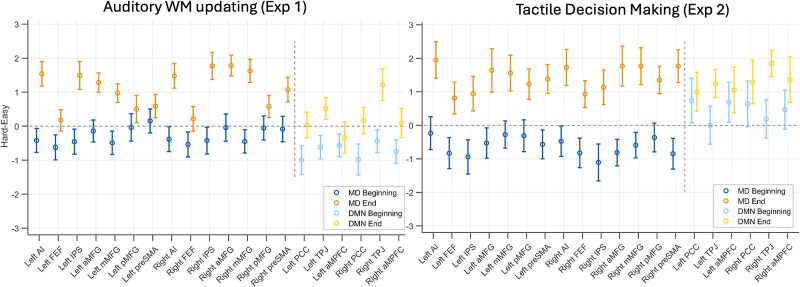
Estimates of activity at the beginning showed a relative deactivation for hard compared to easy episodes, even when only the beginnings and ends of episodes were modeled, and the main episode duration was left unmodeled. While the size of this relative deactivation decreased, hard–easy estimates remained negative in nearly all ROIs. This is in sharp contrast to these estimates at the end of the episode, which were sharply positive. Error bars represent 95% confidence intervals. MD ROIs include anterior middle frontal gyrus (aMFG), middle middle frontal gyrus (mMFG), posterior middle frontal gyrus (pMFG), frontal eye fields (FEF), pre-supplementary motor area (preSMA), intraparietal sulcus (IPS), and anterior insula (AI). DMN ROIs include posterior cingulate cortex (PCC), anteromedial prefrontal cortex (aMPFC), and temporoparietal junctions (TPJ).

Figure 8 shows that the beginnings of hard episodes still exhibited a relative deactivation compared to those of easy episodes; in contrast, at completions, hard episodes elicited a relative activation. Combined across all MD ROIs, the hard–easy difference was still negative at the beginning for both experiment 1 (BF_10_ = 8) and experiment 2 (BF_10_ = 27,505). These effect sizes were smaller compared to our original analysis. But this is to be expected because, in this new model, the unmodeled main episode duration–related activity, which is in the opposite direction to the beginning-related activity (and shows relative activation during hard episodes), will also get captured by the beginning regressors.

Thus, the deactivation at the beginning could not be due to the simultaneous modeling of the episode with event and epoch regressors that capture the initial and sustained activations. It was present even when only a beginning, event regressor was present, and the main episode duration was left unmodeled. To further confirm the issue of deactivation at the beginning of episodes, we include an experiment with a very different task design, where the first step was well separated from subsequent steps, and where deactivation could be documented without making assumptions about the hemodynamic basis function.

### Experiment 3

**Figure 9:**
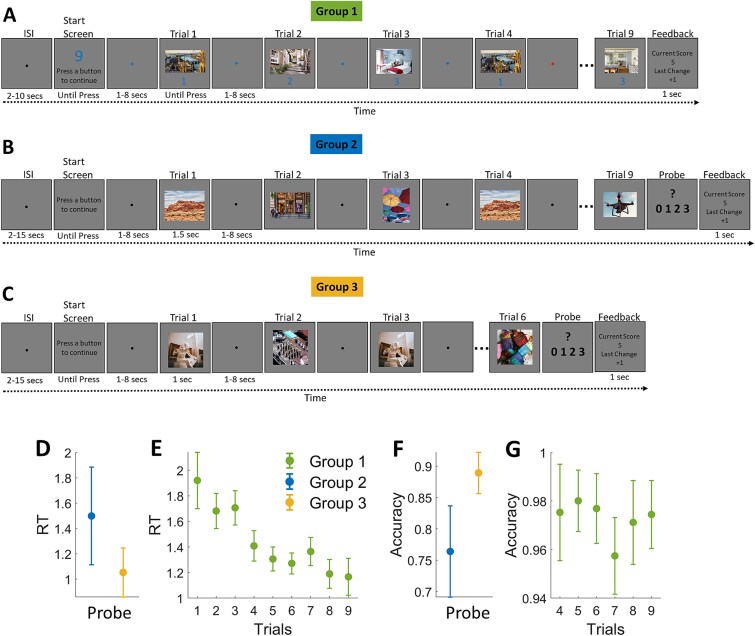
(Experiment 3) All three groups executed trial sequences that began with a start-screen and ended with a feedback screen conveying their performance on the preceding sequence. Individual trials were separated by a jittered 1- to 8-s interval. (A) Group 1 executed a sequence of nine 3-back trials. Each trial stimulus remained on until a response was made, indicating whether the current stimulus was a repeat from 3 trials earlier. (B) Group 2 executed a 9-trial sequence of 3-back trials, but instead of responding on each trial, they were to covertly detect 3-back repeats and keep a count. They had to convey the number of these repeats to the probe at the end of the sequence. These requirements forced group 2 participants to execute the sequence as a single task unit. Furthermore, other demands were higher in group 2 than in group 1 because they required detecting repeats within a limited time, keeping a count of these repeats, and maintaining this information despite interference from subsequent task execution. In contrast, group 1 participants were only to detect a repeat and respond by pressing a button, and they could take as long as they wanted for this. (C) Group 3 participants executed episodes identical to those of group 2 but detected and counted 2-back repeats. This means they had to keep in mind and update only 2 pictures instead of 3. (D) Group 2 participants took longer to respond to the probe and were more error-prone (F). RTs (E) and accuracies (G) of group 1. Error bars represent 95% confidence intervals.

In experiment 3, we used a very different design (Fig. 9). Group 1 executed a sequence of 9 relatively independent 3-back trials, in which participants responded when the current stimulus (picture) matched that 3 trials ago. Group 2 executed a similar sequence of nine trials, but they were cohered into a single episode because they were steps to an overarching task: counting the number of 3-back repeats. Importantly, the task for group 2 was more demanding than group 1. Group 2 had to keep in mind the number of repeats detected so far and maintain and update this information as they separately encoded and updated the memory of the three previous pictures while deciding the repeat status of the presented picture. While group 1 could take as much time as they wanted on any individual trial before encoding, updating, and deciding, for group 2, these had to be done within 1.5 s. As evident in Fig. 9E, on initial trials, group 1 took longer than 1.5 s, suggesting that encoding pictures on these trials would have been additionally demanding for group 2.

Lastly, the stimulus pictures for group 1 were selected from a pool of 89 pictures. This made the chances of a picture repeating on any position other than the 3-trial repeats very low (probability ~0.1). Hence, whenever group 1 participants saw a picture recur within a sequence, it was likely a 3-back repeat, and so these participants did not have to stringently remember picture positions, nor did they face interference from relevant pictures repeating at irrelevant positions. In contrast, pictures for group 2 were drawn from a much smaller pool of 22, and the chance of a picture repeating at positions other than the 3-back repeat was high (probability ranged from 0.3 to 0.4). This forced the participant not only to remember picture identities but also to closely monitor their positions and to contend with high interference from relevant pictures that repeated at irrelevant positions. Error rates further proved that demands were much higher for group 2 (Fig. 9F and G).

Thus, not only were the sequences executed by group 2 more cohered as a single episode but they were also more demanding and required a more complex set of control processes. The program instituted at the beginning of the episode for group 2 can be expected to be more complex and demanding than that for group 1. It can therefore be expected that group 2’s episodes will begin with lower activation or deactivation than group 1’s episodes.

We also had a group 3, who executed episodes identical to group 2, except that they had to covertly detect and keep a count of 2-back repeats over 6 trials, unlike group 2’s 3-back repeats over 9 trials. This group thus had to maintain and update only two previous pictures over a shorter episode. Since the episode executed by group 3 was less demanding than that of group 2, the program instated at the beginning can also be expected to be less complex and cause less deactivation.

**Figure 10:**
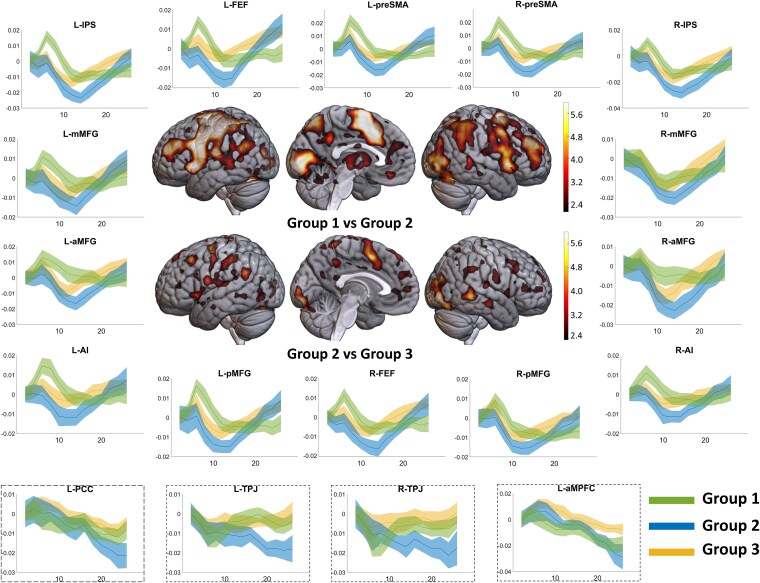
(Experiment 3) Plots show the time course of activity from the beginning of the trial sequences over the ensuing 26 s for the 3 groups in the MD and DMN regions. Note that while sequences for group 1 started with an activation in all MD regions, those for group 2 began with a deactivation. But in group 2, activity subsequently ramped up to a higher level than in group 1. The deactivation elicited in MD regions in group 2 was also higher than in group 3. Shaded ribbons represent 95% confidence intervals. Brain renders show regions where the effect of time on activity interacted with the group in whole-brain analysis (FDR-corrected *P* < .05): Top, group 1 vs. group 2; bottom, group 2 vs. group 3. MD ROIs: anterior middle frontal gyrus (aMFG), middle middle frontal gyrus (mMFG), posterior middle frontal gyrus (pMFG), frontal eye fields (FEF), pre-supplementary motor area (preSMA), intraparietal sulcus (IPS), and anterior insula (AI). DMN ROIs: posterior cingulate cortex (PCC), anteromedial prefrontal cortex (aMPFC), and temporoparietal junctions (TPJ).

Figure 10 shows the activity elicited time-locked to the beginning of the sequence of the three groups. Across all MD regions, the beginning of the sequence in group 1 elicited an activation (Fig. 10, green plots), but in group 2, it elicited a deactivation (blue plots). Furthermore, group 2’s time course of activity ramped up more after this initial deactivation and ended up higher than in group 1. To statistically test for this differential effect of time, we did a Bayesian mixed-effects ANOVA with group, time, and ROIs as fixed factors and subject as a random factor. Group × time interaction was highly significant for MD regions (BF_incl_ = 2 × 10^178^). A whole-brain repeated-measures ANOVA looking for interaction between group and time again highlighted the MD regions (Fig. 10, whole-brain render, upper row). Group 3, who executed a sequence that was structured identically to group 2 but was less demanding, showed lesser deactivation in all of their MD regions compared to group 2 (yellow vs. blue plots). Again, group × time interaction was significant across MD regions in ROI analysis (BF_incl_ = 4.9 × 10^26^).

In summary, the same conceptual result as experiments 1 and 2—MD deactivation at the beginning of demanding episodes—was observed in a very different experimental design, suggesting that the phenomenon may be very general and reflect a domain-general demand that comes into play at the beginning of extended task episodes. It was shown by all MD regions and the effect did not differ between the cingulo-opercular (AI and preSMA) and frontoparietal (MFG and IPS) subsets (BF_incl_ = 0.002). It is noteworthy that DMN regions, which are well known to deactivate during task execution, showed a very different pattern from MD regions. While for group 2, MD regions showed an initial deactivation followed by a ramp-up, DMN regions showed a delayed deactivation that gradually deepened across the episode (Fig. 10, inset plots). The time courses of activity across the different groups were also significantly different across the four DMN regions we analyzed (group 1 vs. group 2, BF_incl_ = 8.23 × 10^8^; group 2 vs. group 3, BF_incl_ = 4.45 × 10^12^).

### Experiment 4

Here, we made a direct test of our thesis against existing accounts by making the first step of difficult episodes have a significantly higher WM demand than the first step of easy episodes. Since WM load is a well-known activator of MD regions, this pits the phenomenon of MD regions deactivating at the beginning of hard episodes against that of their well-known activation to WM demands. If the MD deactivation at the beginning of difficult episodes is a stronger effect, then step 1 of these episodes will elicit a relative deactivation despite having a higher WM demand.

**Figure 11:**
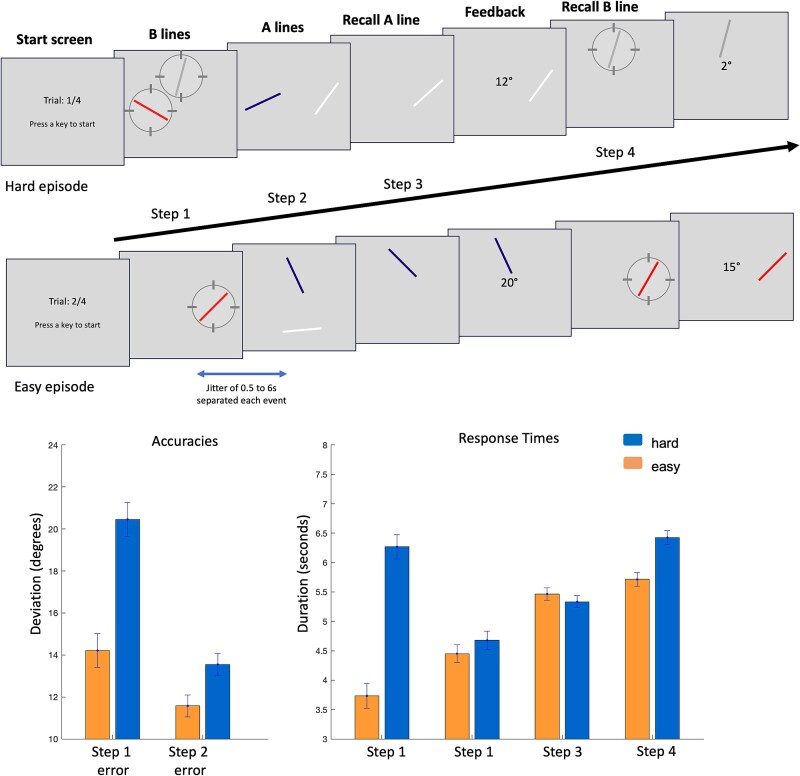
(Experiments 4 and 5) Participants executed two kinds of extended task episodes. Hard episodes began with the presentation of two lines whose orientations were to be kept in mind for subtask-B (step 1). They then (step 2) saw two different lines (referred to as subtask-A lines), kept their orientations in mind, and were probed to recall the orientation of one of these by orienting the probe to the same angle (step 3). After finishing this subtask-A, they were probed to recall the orientation of one of the subtask-B lines (step 4). Easy episodes were identical to hard episodes except that they started with a single subtask-B line presentation. Hard and easy episodes thus differed in the number of subtask-B lines that were to be kept in mind across the duration of the episode while subtask-A was being executed. Recall accuracies were poorer in hard episodes at both steps 3 and 4. Participants took longer to register line orientations on step 1 and took longer to recall these orientations on step 4 of hard episodes. These times for steps 2 and 3 did not differ significantly across hard and easy episodes. Error bars represent 95% confidence intervals.

The experiment design described below was also used in experiment 5. Participants did an extended task consisting of four steps, organized as two interleaved subtasks (Fig. 11). They first saw a start-screen that asked them to press a button to start the episode. During easy and hard episodes they saw one and two lines, respectively (hence referred to as subtask-B lines), whose orientation they had to keep in mind. These stayed on until a button was pressed. Participants then saw two other lines (subtask-A lines) whose orientations were also to be kept in mind (step 2). These, too, stayed on until a button was pressed. Participants were then probed to recall the orientation of one of the subtask-A lines by orienting the probe to the same angle (step 3). Lastly, they were probed to recall the orientation of one of the subtask-B lines, presented during step 1, by orienting the probe (step 4). Easy and hard task episodes required that the orientation of one and two subtask-B lines, respectively, presented on step 1, be kept in mind until the end of the episode (step 4) while shielding them from the interference of subtask-A lines. Each step began 0.5 to 6 s after the response to the previous one. Along with the variability added by response times (1 to 10 s), this allowed us to estimate the activity following each of the step onsets individually without making assumptions about the nature of the hemodynamic basis function.

Since hard episodes involved maintaining a greater number of line orientations in WM and a more complicated set of operations to keep subtask-A lines from interfering with subtask-B lines, their step 1 will be expected to involve instantiating a more complex program. But step 1 of these episodes also involves encoding a higher WM load, which is known to activate MD regions. The current experiment, thus, pits these two effects against each other. If instantiating more complex programs at the beginning of hard episodes deactivates MD regions, then MD regions should deactivate at step 1 of hard compared to easy episodes. By step 2, the program is already in place, and hard episodes require more complex WM operations (instantiated through this program) to keep line representations related to the two subtasks from interfering. Hence, step 2 of hard episodes should show greater MD activation. In contrast, if activation to WM demands was the stronger effect, then MD regions should activate at both steps 1 and 2 of hard episodes compared to easy episodes, due to increased WM demands at both steps.

Since the design was identical for experiments 4 and 5, we combined their behavioral results. Participants expectedly took longer to register subtask-B lines at step 1 of the hard episodes than at step 1 of the easy episodes, and they took longer to recall these lines at step 4 of the hard episodes (Fig. 11; *t*_59_ > 6, *P* < .001, for both). However, these times did not differ at steps 2 and 3 of the easy and hard episodes (*t*_59_ < 1.46, *P* > .15 for both). Recollections were more erroneous during hard episodes for both subtasks B- and A-line orientations (*t*_59_ > 3.7, *P* < .001, for both). The duration participants waited at the start-screen did not differ between the easy and hard episodes (*t*_59_ = 0.01, *P* = .99).

**Figure 12:**
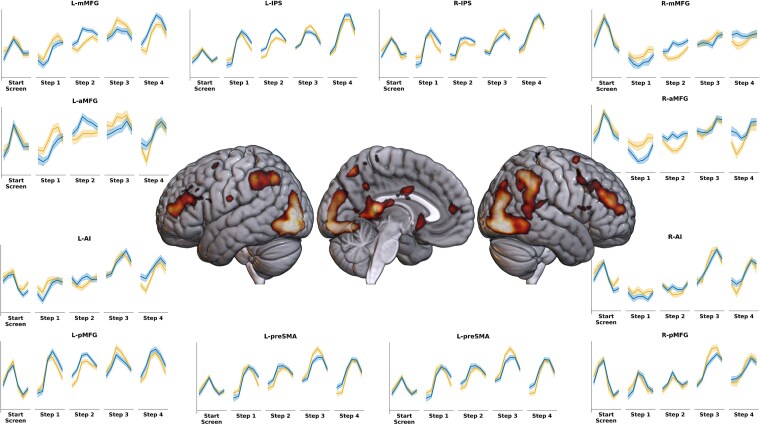
(Experiment 4) Whole-brain render shows regions where the effect of difficulty, calculated as the difference between the average of 6 FIR regressors modeling the step, differed across steps 1 and 2 (FDR, *P* < .05). Plots show the time series of activity across 12 s following the onset of the start-screen and the 4 subsequent steps. In many MD regions, hard step 1 elicited lower activity than easy step 1, whereas hard step 2 elicited higher activity than easy step 2. Shaded regions depict 95% confidence intervals.

Plots in Fig. 12 show a time series of activity across 12 s from the start of a step (as well as the start-screen). We focused on steps 1 and 2. Activity in most MD regions was lower at step 1 of hard episodes than at step 1 of easy episodes. In contrast, at step 2, the activation was higher during hard episodes in most regions. We compared responses in MD regions across steps 1 and 2 for the two kinds of episodes using a Bayesian ANOVA with steps (1 and 2), difficulty (easy and hard), time (FIRs 1 to 6), and ROIs as fixed factors and subject as a random factor. The effect of difficulty was significantly different across the two steps (BFincl = 10^15^). Cingulo-opercular and frontoparietal subsets did not differ in this regard (BFincl = 0.002).

The brain render in Fig. 12 shows the results of a whole-brain analysis (repeated-measures ANOVA) looking at regions whose response to difficulty (estimated as a contrast between the average activity across the six FIR bins of hard vs. easy episodes) differed at steps 1 and 2. Key MD regions—bilateral middle frontal regions (their anterior part and middle parts) and the inferior bank of the IPS (extending into the inferior parietal lobule)—along with parts of the DMN like the temporoparietal junctions (bilaterally but more on the right), as well as the visual cortices, showed a differential effect of difficulty across steps 1 and 2. Results were identical when steps were modeled not with FIR regressors but only their onsets were modeled with event regressors of zero duration (Fig. S1).

Thus, even though step 1 of hard episodes involved a higher WM load, MD regions still deactivated during it compared to step 1 of easy episodes, and these same regions activated during step 2 of these hard episodes. A categorically different response of MD regions to difficult task episodes at steps 1 and 2 is again evidence that these positions involve categorically different issues. It is noteworthy that the effect of difficulty not only switched across steps 1 and 2, they also switched across steps 3 and 4. In some ROIs, the effect of difficulty on step 3 was like that on step 1, with easy episodes showing greater activity than hard episodes. Likewise, the effect of difficulty on step 4 was similar to that on step 2, with hard episodes showing greater activity than easy ones. This was not predicted by us. However, we speculate that this may be due to steps 1 to 4 being organized into two subepisodes. Steps 1 and 2 involve encoding line orientations; steps 3 and 4 involve recollections. It is likely that the task episode is chunked into two distinct subepisodes, one for encoding and one for recall. As a result, step 3 starts a new subepisode and shows the same difficulty effect as step 1, with the easy condition having higher activity than the hard condition. Likewise, step 4 shows the same effect as step 2, with the hard condition having higher activity.

### Experiment 5

Control interventions also increase pupil size (Mathôt 2018; van der Wel and van Steenbergen 2018; Strauch et al. 2022). If instating episode-related programs at the beginning has a distinct neural signature compared to that of control processes later in the episode, then it may also have a distinct psychophysiological signature. We used the same design as in experiment 4. Since WM demands are also well known to increase pupil size (Kahneman 1973; Unsworth and Robison 2018), step 1 pits the effects of WM demand against that of instating the program. Step 2, in contrast, involves only WM demands.

**Figure 13:**
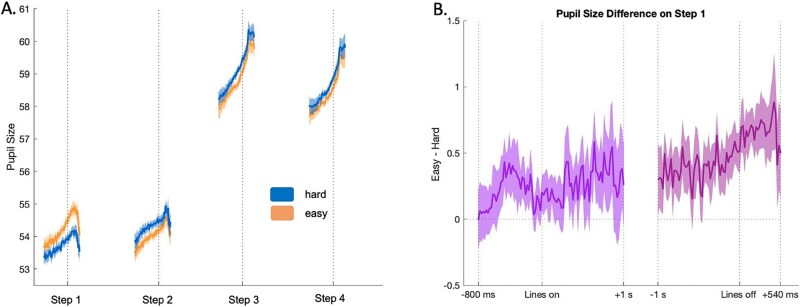
(Experiment 5) (A) Time series of pupil size across the 4 steps of the hard and easy task episodes. Line plots represent the mean values, and shading shows the 95% confidence intervals. Dotted vertical lines represent the time when participants pressed the button marking the completion of the respective step. Plots show pupil size 1 s before and 500 ms after this completion. (B) Time series of pupil size difference between easy and hard step 1 (easy–hard) during different parts of step 1. The left plot (violet) shows the time series of this difference from 800 ms before to 1 s after the onset of lines during step 1. Note that the difference between easy and hard step 1 s increases immediately *prior* to the onset of these lines but decreases after their onset. The plot on the right (lilac) shows the time series of the difference from 1 s before to 540 ms after the offset of lines on step 1. Note that the difference increases immediately *after* the offset. Both show that the pupil size difference cannot be driven by the visual differences between easy and hard step 1 because if such were the case, then the difference should have been higher while the lines were on and not prior to their onset and after their offset.

As evident in Fig. 13A, at step 1, pupil size decreased during hard compared to easy episodes despite the former involving a higher WM load. At step 2, in contrast, hard episodes had a larger pupil size. Crucially, as with MD activity, the effect of difficulty at steps 1 and 2 was significantly different (BF_incl_ = 10^79^). Thus, paralleling MD deactivation, step 1 of hard episodes had a smaller pupil size.

Although the easy and hard screens at step 1 had equal illumination, it is still possible that the requirement to focus on an additional colored line during the hard step 1 led to a decrease in pupil size. However, if this or any other visual issue resulting from the presence of an additional line during hard step 1 was the cause of decreased pupil size, the pupil size difference between easy and hard conditions would primarily be present while the lines were on, and the difference would be absent before the onset and after the offset of these lines. But, as evident in Fig. 13B, the opposite was the case. The difference between pupil sizes on step 1 of easy and hard episodes was larger immediately before the onset of lines and immediately after their offset. Pupil sizes began to differ before step 1 onset because, in most instances, participants could anticipate the nature (easy or hard) of the ensuing episode (see Methods). The difference remained throughout step 1 and increased further after the lines disappeared. The decreased pupil size on step 1 of hard episodes cannot, therefore, be due to visual issues resulting from the presence of an additional line. This potential confound also gets ruled out by the observation that participants who showed a greater decrease in their pupil size on step 1 of hard compared to easy episodes were more likely to show a greater deviation in their responses on step 4 of hard compared to easy episodes (Kendell’s tau = 0.29, BF_10_ = 5).

## Discussion

Activation and deactivation to cognitive demands are well known but have been thought to involve distinct sets of regions, ie MD and DMN regions, respectively (Raichle et al. 2001; Fedorenko et al. 2013). Activation of MD regions to diverse control demands is a key finding in neuroscience, forming the basis for the idea that different tasks may involve common, domain-general task demands (Duncan 2010; Assem et al. 2020; Fedorenko and Blank 2020). We showed that the *beginning* of difficult task episodes deactivates these same regions and decreases pupil size. These demand-related MD deactivations were observed across very different task episodes, including auditory WM updating, visual working memory interference, and tactile perceptual judgment, suggesting that they may reflect domain-general demands that occur at the beginning of diverse extended task episodes, regardless of content or structure. But since these demands are related to the overarching episode, and not to individual steps, they may be higher-level than those that occur within the episode and activate MD regions and increase pupil size.

This deactivation at the onset of more difficult episodes occurred across all MD regions, and no functional distinction was evident among them. There has been a suggestion that the cingulo-opercular (preSMA and AI) components may be functionally distinct from the frontoparietal ones (MFG and IPS). The cingulo-opercular component has been suggested to initiate tasks and maintain goals, while the frontoparietal component is thought to instantiate control of attention and working memory (Crittenden et al. 2016; Dosenbach et al. 2006, 2025; Sridharan et al. 2008a). We did not find any distinction in the responses of these two sets of regions.

Unlike MD regions, DMN regions’ response to demands at the beginning versus during the main episode was inconsistent (Figs. 7 and 10). In experiment 1, they showed a relative deactivation at the beginning of hard episodes, but in experiment 2, they showed a relative activation. In experiment 3, core DMN regions, PCC, and AMPFC showed no deactivation at the beginning of more demanding episodes; the deactivation was delayed and then deepened across the episode. This was in sharp contrast to MD regions, which showed initial deactivation followed by a ramp-up.

### Hierarchical cognition

Beginning extended tasks involves creating a hierarchically organized cognition with entities (representations/operations/processes) at multiple levels, with higher-level entities controlling and organizing lower-level ones (Miller et al. 1960; Broadbent 1977; Logan 2004; Shallice and Cooper 2011). Goal-directed programs are fundamental to this, as they are the higher-level entities that serve as the means of controlling and executing extended tasks in relation to the overarching goal (Farooqui and Manly 2018a). This idea of hierarchical organization of cognition during purposive behavior is very different from many existing notions of hierarchical control. Some have conceived of hierarchy in terms of levels across which information is to be integrated before a decision is reached, eg choosing the response in relation to the stimulus versus choosing how the stimulus relates to the response in relation to the larger context and then choosing the response (Koechlin 2003; Duverne and Koechlin 2017). Others have conceived of hierarchy in terms of levels of decision-making needed to reach a response, eg when a stimulus specifies a response versus when a stimulus (or one of its dimensions) specifies how another stimulus is to be used to select a response (Badre and Nee 2018).

Our finding that instating higher-level, episode-related programs was primarily accompanied by widespread deactivations is also very different from the expectations of the accounts of hierarchical control. For a set of them, more anterior regions of the PFC activate to higher-level entities (eg Koechlin 2003; Christoff et al. 2009; Badre and Nee 2018). For others, the DMN may be engaged with higher levels of cognition and may represent temporally extended episodes in cognition (Speer et al. 2007; Margulies et al. 2016; Wen et al. 2020). Some have also suggested that MD regions, especially lateral prefrontal regions, may be the ones that instantiate programs controlling and executing extended sequences of thought and behavior (Duncan 2013; Nee and D’Esposito 2016). In contrast to the current findings, these accounts would, respectively, have predicted that anterior prefrontal regions, DMN, and MD regions would have higher activation at the beginning of more demanding task episodes.

### Episode-related programs

The evidence of these programs at the beginning of diverse kinds of task episodes suggests that their role may be general and applicable to any extended task. All extended tasks require a dynamic organization of cognition across time by continually creating the correct configurational changes in various perceptual, attentional, mnemonic, and motor domains so that at every instant within the task episode, cognition is anticipatorily in the most optimal state achievable for the expected demands (Bartlett and Bartlett 1932; Logan and Gordon 2001; Miller et al. 1960; Nobre and van Ede 2023; Palenciano et al. 2019; Rogers and Monsell 1995). What exactly gets done as part of these may, of course, vary across different tasks.

During, eg a typical block or episode of visuo-motor trials, processing related to mind-wandering, ongoing unconscious goals, task-irrelevant sensory and motor processing, etc. need to be relegated (Aarts and Custers 2012; Cardellicchio et al. 2020); the predictiveness of the block needs to be utilized to make anticipatory changes, eg the knowledge that responses would be right handed, visual attention limited to the area around fixation, along with an implicit idea of inter-trial intervals, will get used to increase attention and make available the correct perceptual and motor processing routines at times when a stimulus is expected and decrease them when inter-trial intervals are expected, etc. (Coull and Nobre 1998; Ghose and Maunsell 2002; Janssen and Shadlen 2005; Cravo et al. 2017; Denison et al. 2017; Los et al. 2017; Zimmermann et al. 2017; Grabenhorst et al. 2019).

Furthermore, cognition does not just use temporal expectations to make such anticipatory set changes but also uses them to make control interventions. Control processes are maintained across time in the way and for the duration that is expected to be most optimal based on task knowledge and past experiences. Thus, at the expected juncture, all kinds of processes get enhanced—accumulation of perceptual evidence and fine-tuning of response threshold (Jepma et al. 2012; Fernández et al. 2019), perceptual speed and acuity (Vangkilde et al. 2012; Fernández et al. 2019), control of WM (Wilsch et al. 2015; Gresch et al. 2021), arousal and cognitive resources (Shen and Alain 2012; Shalev and Nobre 2022), and memory encoding (Jones and Ward 2019), to list a few.

All of these occur seamlessly and automatically once the episode is embarked upon. These very many *metacontrol* changes through which control processes are controlled and organized across time cannot be achieved through separate and independent neurocognitive acts and must be instantiated as part of a single goal-directed program. It is plausible that programs evidenced in this study correspond to configurational changes created at the beginning of the episode that adjust widespread synaptic weights in such a way that, across the ensuing task episode, neural population activity in widespread regions will be channeled through a specific sequence of states corresponding to the relevant sequence of cognitive states. Such task-related configuration of synapses will decrease ongoing but irrelevant neural activity, causing a deactivation that correlates with the length and complexity of the ensuing episode. Completion of episodes may reset these synaptic changes, causing a widespread burst of activity seen at task completions (Farooqui et al. 2012; Fox et al. 2005a; Fujii and Graybiel 2003; Sridharan et al. 2008b). Dismantling more complex programs at the completion of more complex episodes may likely lead to more intense activation at the end of more demanding episodes. This was indeed observed in the current study and provides additional evidence for the presence of overarching programs (Figs. 3 and 6).

There have to be limits on how big/complex a program can be instated or maintained at a time. Consequently, long and complex trial sequences are not executed as one task unit through a massive program; instead, they are chunked and executed as a series of smaller units, each executed through a smaller program (Farooqui et al. 2023). Notably, this occurs even though these trial sequences did not involve WM load and hence this chunking cannot be attributed to WM load. The same is seen with motor sequences. As individual motor acts become easier and less demanding with practice, the length of their sequences that can be executed as a single unit through a single program increases (Ramkumar et al. 2016; Tosatto et al. 2022). This is also suggested by the nature of the task labels people use to describe their behavior (Vallacher and Wegner 1987, 1989). When executing easy and routine behaviors, individuals typically choose labels corresponding to long tasks, eg saying that they are “preparing breakfast” rather than “chopping fruit.” In contrast, when the same behavior in the same context becomes more challenging, eg when using a heavy and blunt knife, they become more likely to use the “chopping fruit” label. When chopping fruit with a very heavy knife, fewer resources are available to maintain large programs for long task units, so behavior is executed through smaller programs as smaller task units.

### Pupil size and programs

None of the existing psychophysiological accounts predict the dichotomous pupil response during hard versus easy episodes, in which pupil size decreased at the beginning but increased subsequently. WM and attentional demands, cognitive conflict, response inhibition, and rule-switching—all have been seen to dilate pupils. This has been variously interpreted as reflecting increased mental load, arousal, attention, effort, etc. at junctures that involve such control demands (van der Wel and van Steenbergen 2018; Strauch et al. 2022). Such explanations, however, cannot account for why task difficulty would cause a decrease in pupil size at the beginning of a difficult episode but an increase during its subsequent execution. For such accounts, pupil size should either have increased at the beginning of difficult episodes or remained unchanged.

A recent popular account has tried to explain task-related changes in pupil size in terms of exploration–exploitation trade-offs and posits that pupil size is greater during periods of exploration, allowing for increased access to the visual periphery and is smaller during exploitation when the ongoing mental task is being focused upon or “exploited,” which allows for more intense but narrow focus (Gilzenrat et al. 2010; Jepma and Nieuwenhuis 2011; Grujic et al. 2024). This is based on the finding that the pupil size tends to be smaller when participants are more on-task and showing higher task performance, and larger when they are less on-task and more mind-wandering. While this account gives a plausible explanation for why pupil size may decrease at the beginning of difficult compared to easy episodes (“more intense task focus or exploitation”), as per its predictions, the pupil size should have remained small on step 2 of difficult episodes because these too required a more intense task focus. The dichotomous response of pupils strongly suggests that categorically different demands are needed at the beginning of difficult task episodes from those needed during their subsequent execution. It is known that pupil size correlates with that of MD regions during demanding tasks (Fietz et al. 2021; Schneider et al. 2016). Whether their dichotomous responses during extended tasks are driven by MD regions’ dichotomous responses remains to be determined.

### Other explanations

Our study reports an empirical finding. We demonstrated its generalized nature by showing its presence across diverse task designs and content. We additionally showed that this phenomenon is not just cortical but also manifests in psychophysiological markers reflecting the activity of brainstem modulatory systems. Interpreting cognitive goings-on from neural activity can never be straightforward. We had predicted the current observations based on previous studies suggesting that instating a higher-level goal-directed program at the start of extended tasks involves a distinct form of demand compared to instantiating control processes later via this program. We interpreted the current results in this framework. As we discuss below, other explanations, though plausible, cannot account for all related findings from the current and previous studies.

Perhaps participants paused longer before beginning difficult episodes, leading to relative deactivation at their beginning. This is unlikely. In all experiments, deactivation was noted *after* participants had begun executing. This is most evident in experiments 3 and 4, in which deactivation occurred when participants were explicitly engaged in demanding tasks, not during the prior waiting period. Furthermore, waiting times before initiating the episode were identical across hard and easy conditions in all experiments.

Another possibility is that initial deactivation was due to a preparatory resetting of neural activity and synaptic weights that prepared the brain for the ensuing task episode, but no program remained active after this resetting; hence, the construct of an ensuing program is unnecessary. While the crux of our argument—episode initiation has distinct demands that deactivate MD regions—would still hold, we do not favor this account because it cannot explain several other episode-related phenomena. After initial deactivation, MD activity does not remain constant; it ramps up across the episode duration (Fig. 10) (see also Farooqui and Manly 2018a; Desrochers et al. 2019). Furthermore, episode completion elicits intense activation that correlates with the episode’s complexity (Figs. 3 and 6) (Farooqui et al. 2012). All of these suggest that the phenomenon at the beginning of the episode is part of something that continues through the episode and wraps up only at completion. We therefore favor the construct of a program that runs from the beginning to the end and subsumes task execution.

Perhaps the initial deactivation is a metabolic preparation for the ensuing activation. The more a brain region needs to activate, the more it deactivates before this activation, allowing for some form of energy-related homeostasis. This possibility, on the surface, appears different from our account, but in reality, it is the same, as it assumes a program or some cognitive entity that takes into account the demands that will arise later during the ensuing episode and decreases the metabolic activity of MD regions (and deactivates them) accordingly. Furthermore, as evident in other studies (Farooqui and Manly 2018a), the rate at which this initial deactivation returns to toward the baseline depends on the duration of the episode and the proportion of the episode executed thus far. This requires that the cognitive entity determining the decrease in metabolic activity track progress in episode execution, another feature that will make this entity a subsuming cognitive program.

## Summary

We showed that activation of MD regions and increased pupil size during difficult tasks is only part of the story. The initiation of these very tasks is accompanied by deactivation of these regions and decreased pupil size, showing that the cognitive demands of initiating difficult task episodes are categorically different from those related to instantiating control interventions later in these episodes. We suggested that the demands at the beginning of difficult episodes pertain to instantiating metacontrol programs that will subsequently goal-directedly organize and cohere the various control interventions during the ensuing task execution.

## Supplementary Material

Supplementary_Figure_bhag046
