# Supplementary material for: Domain-general demands that deactivate multiple-demand regions
Source: Cereb Cortex. 2026 Apr 22;36(4):bhag046. doi: 10.1093/cercor/bhag046 (PMC13099392; doi:10.1093/cercor/bhag046)
Supplement: Supplementary_Figure_bhag046 [file supplementary_figure_bhag046.docx]

**Domain-General Demands that Deactivate Multiple-Demand Regions**

Tamer Gezici^1,2,3*^, Elif Oymagil^1,2,3*^, Adem Yazici^3,5*^, Berhan F. Akgur^1,2,3^, Ipek Çiftçi^1,2,3^, Ausaf A. Farooqui^1,3,4,5^

Author affiliations:

1 Department of Neuroscience, Bilkent University, Ankara, Turkiye

2 Interdepartmental Neuroscience Program, Bilkent University, Ankara, Turkiye

3 Aysel Sabuncu Brain Research Center, Bilkent University, Ankara, Turkiye

4 National Magnetic Resonance Research Center, Bilkent University, Ankara, Turkiye

5 Department of Psychology, Bilkent University, Ankara, Turkiye

* Equal Contribution

Correspondence to: Ausaf A. Farooqui

Department of Psychology, Bilkent University, Ankara, 06800, Turkiye

E-mail: [ausaf.farooqui@bilkent.edu.tr](mailto:ausaf.farooqui@gmail.com)


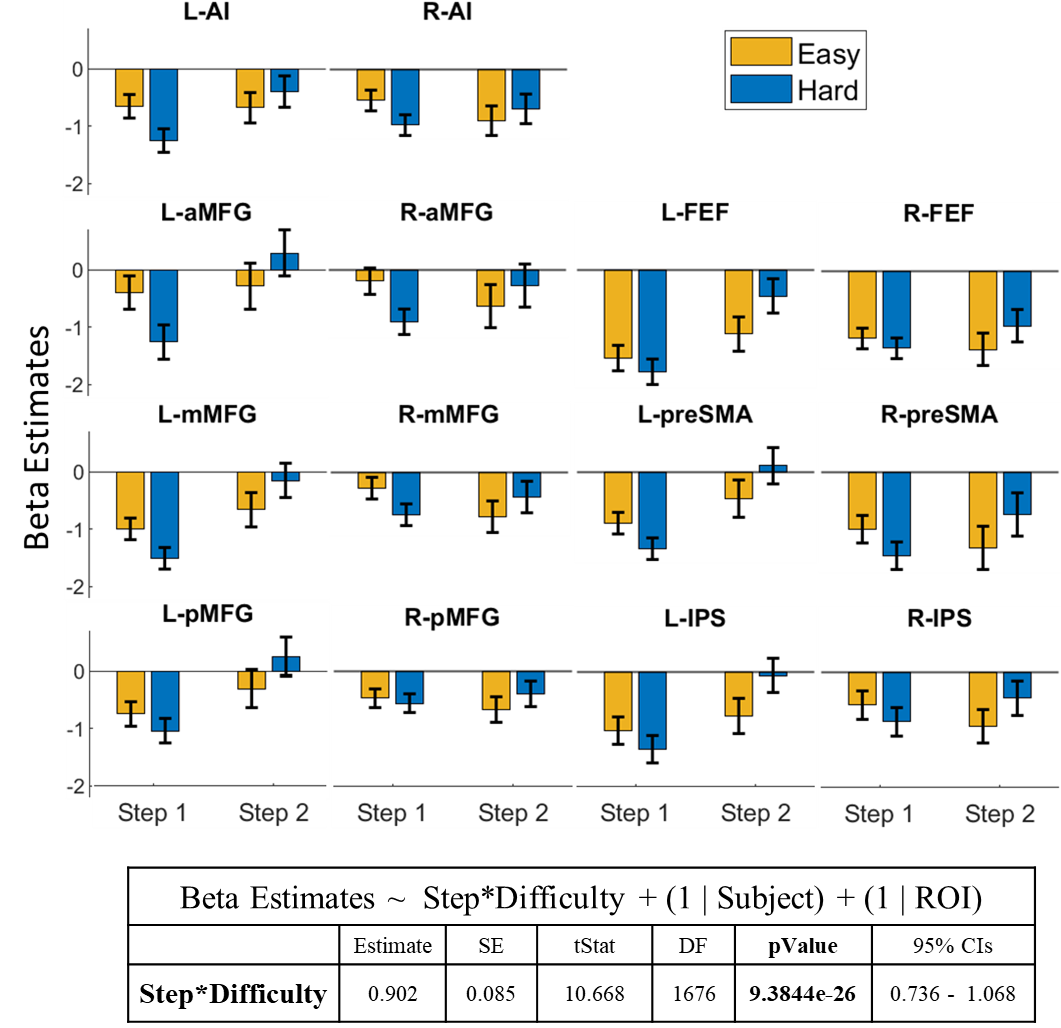


Figure S1. (Experiment 4) The differential effect of difficulty across steps 1 and 2 was seen even when steps were modelled as events of no duration.
